# Long-term follow-up of osteomyelitis caused by Bacille Calmette-Guérin vaccination in immunocompetent children

**DOI:** 10.1038/s41598-020-61152-6

**Published:** 2020-03-05

**Authors:** Yen-Lin Tsai, Yen-Ju Chen, Yu-Cheng Lai

**Affiliations:** 1 Department of Orthopedics, Kaohsiung Veterans General Hospital, Kaohsiung, Taiwan; 2 Department of Marine Biotechnology and Resources, National Sun Yat-Sen University, Kaohsiung, Taiwan; 3 Department of Occupational Therapy, Shu Zen junior College of Medicine and Management, Kaohsiung, Taiwan

**Keywords:** Tuberculosis, Outcomes research

## Abstract

No standard treatment for Bacille Calmette–Guérin (BCG)-associated osteomyelitis, a rare but serious complication of the BCG vaccine, has been established. This study explored the short- and long-term outcomes of surgical intervention for BCG-associated osteomyelitis. Four patients with BCG osteomyelitis aged 9–21 months when diagnosed and treated at the Department of Orthopedics, Kaohsiung Veterans General Hospital, Taiwan, between January 2001 and April 2019 were analysed. Radiography revealed osteolytic lesions of the involved sites. Magnetic resonance imaging revealed physeal involvement in three patients. Debridement was performed for all four patients. One patient then underwent additional arthroscopy because of suspect intra-articular involvement. Specimens obtained intraoperatively were sent for pathology, culture, and to the national reference mycobacterial laboratory for BCG detection using polymerase chain reaction. All four patients had positive results for *Mycobacterium bovis* and satisfactory short-term results. Functional monitoring using QuickDASH or the Lower Extremity Functional Scale revealed excellent long-term outcomes despite tiny limb length discrepancy observed during follow-up in two patients. Prompt diagnosis plus proper intervention is required to prevent further major complications of BCG osteomyelitis. Minimally invasive debridement led to positive clinical outcomes and is therefore recommended.

## Introduction

Tuberculosis (TB) ranks among the top 10 causes of death and is the leading cause of death from infectious diseases worldwide. This disease poses a particularly severe threat in developing countries^1^. Key risk factors include poverty, overcrowding, malnutrition, alcohol and tobacco use, diabetes, and immunocompromised status. Among children, the very youngest ones are at the greatest risk of developing active TB following primary infection^2^.

The Bacille Calmette-Guérin (BCG) vaccine was first used in 1921 and has since been used globally^3^. While this live-attenuated vaccine has abundant safety records and well-documented protective effects against meningitis and disseminated TB in young children^4–6^, several BCG-vaccine-associated complications have long been subjects of discussion; these complications include local adverse reactions such as regional lymphadenitis, injection site abscesses, persistent injection site reactions, ulceration and keloid reactions, and systemic adverse reactions such as osteomyelitis or osteitis and disseminated BCG disease.

Of aforementioned adverse events, BCG osteomyelitis is relatively rare. This disease often begins with insidious, nonspecific symptoms that sometimes result in delayed medical consultation. Although BCG osteomyelitis is well known for its good prognosis, lesions involving vertebrae and the physis of long bone can lead to permanent damage. Late diagnosis leads to extensive damage and accounts for severe complications^7^; moreover, the unavailability of molecular tests for BCG renders definite diagnosis difficult. A standard management for treating this well-documented but rare condition has yet to be established.

However, an invasive procedure has been well-documented to avoid due to the possible disadvantages. This study investigated cases of patients who had undergone debridement for BCG osteomyelitis and provides details concerning the manifestation of BCG osteomyelitis in these cases and their short- and long-term outcomes.

## Methods

We reviewed charts of patients treated at Kaohsiung Veterans General Hospital, Taiwan from January, 2001 to April, 2019 and excluded those without a detailed clinical information. Finally, four patients (3 boys and 1 girl) with diagnoses of BCG osteomyelitis or BCG-associated osteomyelitis or osteitis are enrolled in our study. All methods were carried out in accordance with relevant guidelines and regulations. All experimental protocols approved by Kaohsiung Veterans General Hospital Institutional Review Board. Informed consent was obtained from all subjects and their parents.

The boys were 9, 13, and 14 months old, and the girl was 21 months old when diagnosed. All involved sites were distal section of long bones, including the metaphysis and physis of the right distal fibula, the physis and metaphysis of the right distal ulnar, the epiphysis and metaphysis of the right distal femur, and the epiphysis and metaphysis of the left distal tibia. (Table 1).

**Table 1:** Characteristics of the patients in our study.

No.	Gender	Inoculation site	Age at vaccination	Age at onset	Age of diagnosis	Lesion site	Physis involvement	Clinical manifestation
1	M	Left arm	6.5 m	13 m	14 m	Left distal tibia	−	Swelling and local heat around ankle joint Decreased stepping
2	M	Left arm	1w	9 m	9 m	Right distal ulnar	+	Persistent painless swelling over right wrist for 1 week, fever 38.3’C
3	M	Left arm	1 m	14 m	16 m	Right distal femur	+	Limping gait for 1 months Flexion contracture of right knee
4	F	Left arm	1 m	20 m	21 m	Right distal fibula	+	Swelling & pain of right ankle Unsteady gait

The patients’ initial symptoms varied. Three patients, whose lesion sites were at the lower extremities, exhibited limping gait. One of these exhibited painful disability with range of motion limitation and joint contracture, one had swelling with pain and the other had no swelling or pain.

All the patients were in generally stable condition upon initial presentation, except for one whose lesion was at the right distal ulnar, had a with fever up to 38.3 °C. This patient was a full-term, initially healthy baby who received BCG vaccination within 1 week of birth and was subsequently admitted for painless swelling over the right wrist persisting for more than 1 week.

## Results

### Radiology and image findings

**Figure 1:**
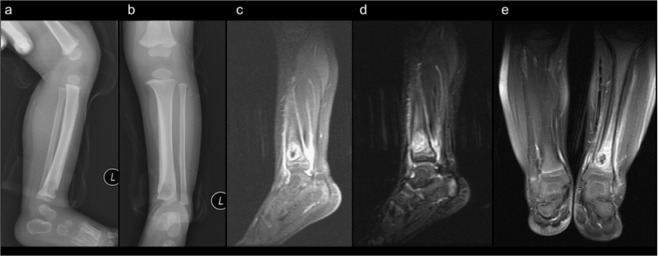
Osteolytic lesion in the metaphysis with cortical destruction and significant periosteal reaction of the left distal tibia (**a**: lateral view, **b**: anteroposterior view). Focal high T2WI signal lesion at the left lower tibia, measuring approximately 1.8 cm with significant periosteal reaction and sinus tract formation alongside soft tissue edema, increased joint effusion, synovial proliferation, and synovitis at the left ankle (**c–e**).

Patient 1’s radiography revealed an osteolytic lesion in the metaphysis of the left distal tibia with cortical destruction and marked periosteal reaction (Fig. 1a,b). Magnetic resonance imaging (MRI) revealed evident bone marrow edema over the left middle and lower tibia at the diaphysis and metaphysis levels. A focal high T2WI signal lesion at the left lower tibia, measuring approximately 1.8 cm in size with significant periosteal reaction and sinus tract formation was observed. Soft tissue edema, increased joint effusion, synovial proliferation, and synovitis at the left ankle were also seen (Fig. 1c–e).

**Figure 2:**
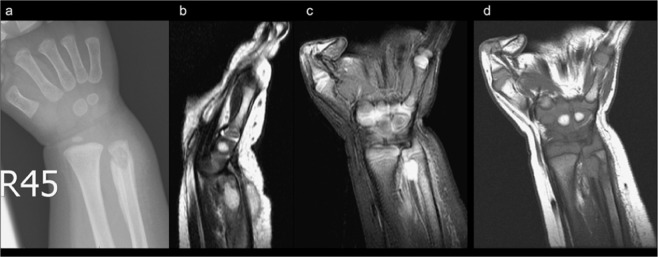
Well-defined osteolytic lesion in the right distal ulnar with cortical destruction. (**a**) Marked solid and thickened periosteal reaction at the right distal ulnar with focal eccentric marrow replacement lesion in the metaphysis region alongside the presence of an inflammatory mass lesion. Dorsal aspect of the right distal forearm that communicates to the nearby bone lesion through a wide sinus tract; surrounding diffuse soft tissue swelling is also noted (**b–d**).

Patient 2’s X-Ray revealed a well-defined osteolytic lesion in the right distal ulnar with cortical destruction (Fig. 2a). This patient’s MRI indicated solid and thickened periosteal reaction at the right distal ulnar with a focal eccentric marrow replacement lesion in the metaphysis region as well as an inflammatory mass lesion. At the dorsal aspect of the right distal forearm, which communicated with the nearby bone lesion through a wide sinus tract (4 mm), surrounding diffuse soft tissue swelling was noted (Fig. 2b–d). Moreover, an active lesion in the bone of the right forearm was observed in the patient’s whole-body bone scan.

**Figure 3:**
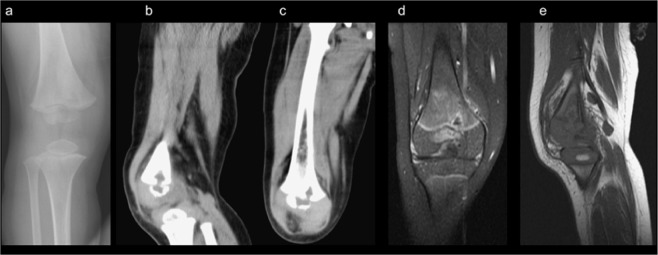
Ill-defined osteolytic lesion in the epiphysis and metaphysis of right distal femur with cortical destruction. (**a**) Focal bone destruction with osteolytic lesion at the metaphysis and epiphysis of the distal femur and irregular thinning and cortex destruction at the ossification centre of the epiphysis, with more in the medial aspect. Obvious synovial proliferation and soft tissue enhancement at the right knee joint was noted, with more in the posterior aspect of the intercondylar notch (**b–e**).

Patient 3’s X-ray revealed an ill-defined osteolytic lesion in the epiphysis and metaphysis of the right distal femur with cortical destruction (Fig. 3a). In the MRI, focal bone destruction with an osteolytic lesion in the metaphysis and epiphysis of the distal femur and irregular thinning and cortex destruction at the ossification centre of the epiphysis, appeared primarily in the medial aspect. Evident synovial proliferation and soft tissue enhancement at right knee joint mainly in the posterior aspect of the intercondylar notch were noted (Fig. 3b–e).

**Figure 4:**
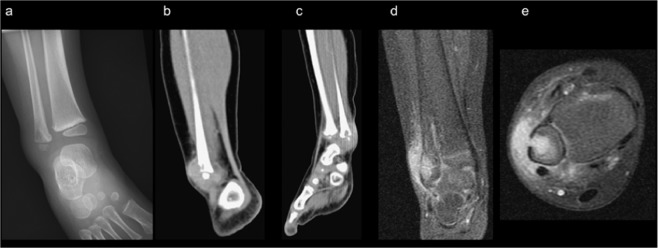
Lytic lesion in the metaphysis of the right distal fibula alongside cortical destruction and a partial osteosclerosing rim. (**a**) Osteolytic bone lesion in the metaphysis abutting to the physis surface of the right distal fibula and focal lucent bone density of the epiphysis of the distal fibula alongside an adjacent swelling inflammatory mass with peripheral enhancement, measuring approximately 2.7 × 2.6 × 1.6 cm on a computed tomography scan (**b,c**). Bone marrow edema in the right distal fibula, surrounding soft tissue swelling, and intraosseous fluid collection with a drainage sinus tract to the extraosseous region, measuring approximately 1.7 × 1.5 × 1.2 cm in magnetic resonance imaging (**d,e**).

As for Patient 4, a lytic lesion in the metaphysis of the right distal fibula with cortical destruction and a partial osteosclerotic rim were observed. (Fig. 4a) In the patient’s computed tomography scan CT, an osteolytic bone lesion in the metaphysis abutting to the physis surface of the right distal fibula was observed as well as focal lucent bone density of the epiphysis of the distal fibula alongside an inflamed mass with peripheral enhancement measuring approximately 2.7 × 2.6 × 1.6 cm in size. (Fig. 4b,c) This patients’ MRI revealed bone marrow edema in the right distal fibula, surrounding soft tissue swelling and intraosseous fluid collection with a drainage sinus tract to the extraosseous region measuring approximately 1.7 × 1.5 × 1.2 cm in size. (Fig. 4d,e).

### Laboratory tests and TB evaluation

The laboratory tests revealed that none of the patients had leukocytosis, including the one who presented with fever. In addition, their C-reactive protein (CRP) levels and erythrocyte sedimentation rates (ESR) were all within the normal range. Chest radiographs of all patients were evaluated and none of them showed lesions of pulmonary TB or contact history of TB.

### Intervention and diagnosis

All of the four patients underwent debridement of their lesion sites. Surgery was arranged for patients 1, 2, and 4 because a connection between a bone cavity and peripheral tissue was observed in the MRI of each, leading to diagnoses of bone abscesses. (Table 2).

**Table 2:** Procedures, pathological findings, medical treatment, and clinical outcomes.

No.	Operation	Pathologic finding	Culture/AFS	Caseous necrosis	Tissue MTB PCR	Medication/total duration	Clinical outcome
1	Debridement	Bony tissue with acute and chronic inflammatory cell infiltration, bone necrosis, and necrotizing granulomatous reaction	+/−	+	+	HR/12 m (ongoing)	Good
2	Debridement	Suppurative granulomatous inflammation	+/+	+	+	HR/10 m	Good
3	Debridement & arthroscopy with synovectomy	Chronic granulomatous inflammation composed of epithelioid cells aggregation	−/−	−	+	HR/12 m	Good
4	Debridement	Granulomatous inflammation with neutrophil infiltration	+/+	−	+	HRZ/1 m HR/12 m	Good

Patient 3, who had a lesion over the right distal femur and joint involvement, received open debridement and arthroscopy with synovectomy of the right knee. Pathology, acid-fast stain, and culture specimens were obtained during the surgery, and all resultant specimens were sent to the National Reference Mycobacterial Laboratory for BCG detection.

The patients’ pathology reports reveled typical granulomatous inflammation morphology with or without caseous necrosis. Acid-fast-stained bacilli were detected in two patients’ specimens. None of the patients had a positive result for Periodic acid-Schiff (PAS) staining.

All four patients’ bacterial culture results were negative. Three of the patients had positive result of the Mycobacterium Tuberculosis complex (MTBC); one had a negative MTBC culture. The specimens of all the four patients sent to the National Reference Mycobacterial Laboratory were confirmed positive for Mycobacterium bovis through polymerase chain reaction (PCR).

### Medical therapy and outcomes

All of the patients received anti-TB pharmacotherapy under the supervision of pediatricians after diagnosis. Apart from the first patient, who was still taking anti-TB agents, the patients had all completed their treatment courses; they had each received a combination of isoniazid and rifampicin for at least 10 months. The third patient had received the isoniazid (H) and rifampin (R)(HR) regimen for 12 months. The fourth patient had taken the isoniazid, rifampicin, and pyrazinamide (HRZ) regimen for 4 weeks before switching to the HR regimen for 12 months.

**Figure 5:**
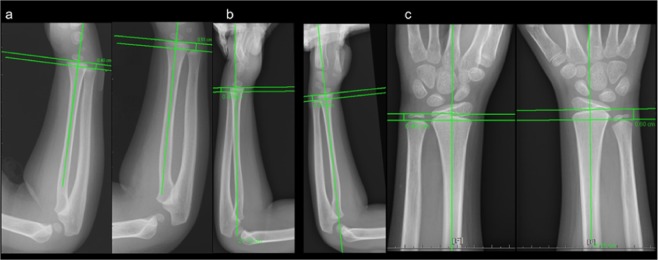
Negative ulnar variances of the left and right hand were 0.4 and 0.51 cm, respectively, 1 year after surgery. (**a**) The right variance was 0.43 cm 1.5 years later, and the left side measured 0.53 cm. (**b**) The right variance was 0.6 cm and the left variance was 0.4 cm 7 years after initial surgery (**c**).

**Figure 6:**
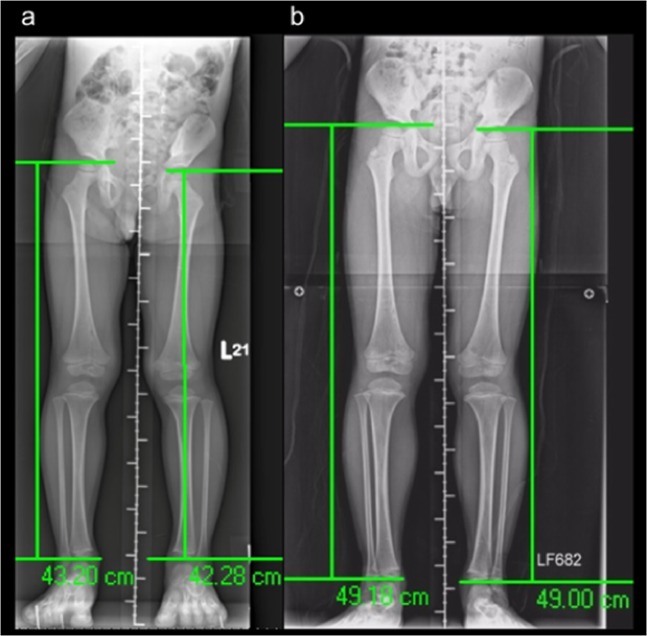
The affected limb was 0.92 cm longer than that on the other side, and the patient had no gait disturbance 28 months after surgery (**a**). Four years after surgery, the discrepancy had decreased to 0.18 cm (**b**).

No residual lesions appeared on the X-rays of any of the four patients after treatment. Nevertheless, limb length discrepancy was observed during follow-up in two of the patients. Forearm discrepancy was measured on posteroanterior radiographs with the wrist in a neutral position. Ulnar variance was defined as the distance between two lines perpendicular to the axis of the radius bone at the level of the most distal edges of the metaphysis in the radius and ulna. The negative variance of the right hand of Patient 2 was 0.51 cm 1 year after surgery, and 0.43 cm another 1.5 years later. The negative ulnar variance was 0.6 cm 7 years after the initial surgery. The negative ulnar variance of the patient’s left hand remained at 0.4 cm throughout the follow-up period. (Fig. 5) In Patient 3, a parallel Harris growth arrest line was observed on X-ray 16 weeks after the surgery. No evident leg length discrepancy was detected by a block test 18 months after surgery; however, another 10 months later, the affected limb was 0.92 cm longer than the corresponding limb on the other side without gait disturbance, as measured by the perpendicular distance between the femoral head and distal tibia. Another 2 years later, 4 years after surgery, the discrepancy had decreased to 0.18 cm. (Fig. 6).

Recent follow-up was conducted at the outpatient clinic of Kaohsiung Veterans General Hospital for Patient 1 and via telephone consultation for the other patients. Patient 2 scored zero points on the QuickDASH questionnaire 7 years after the surgery. The other three patients were evaluated using the Lower Extremity Functional Scale; all demonstrated 100% function. Patient 1 was assessed 6 months after the surgery, whereas the other two patients were assessed 5 years after surgery.

## Discussion

TB is one of the top 10 causes of death and the leading cause of death from infectious diseases worldwide. Younger children are at a higher risk of developing active TB following primary infection. The BCG vaccine plays a major role in protecting young children against severe TB.

Our study analyzed four children with laboratory-confirmed BCG osteomyelitis treated at a single referral centre in Taiwan from January 2001 to April 2019. The most common symptoms were swelling of the bone and soft tissue with mild pain; only one patient presented with fever. All four patients underwent surgical procedures for diagnostic or therapeutic purposes and received antimycobacterial medications for at least 10 months after diagnosis. After treatment, most of the symptoms had improved.

Diagnosis of BCG osteomyelitis is not usually made until the disease has well advanced because of the subtle symptoms^8^. The distinctive features of bacterial osteomyelitis and BCG osteomyelitis and osteitis must be distinguished. Children with bacterial osteomyelitis commonly present with high fever and bone pain of abrupt onset^9^. By contrast, those with BCG osteomyelitis often present with relatively localised symptoms. Of the various symptoms, local tenderness, swelling, and antalgic posturing are the three most commonly reported in the literature^10–12^. The clinical manifestations of our cases were comparable with those of previous studies. Only one child presented with fever, which is an uncommon symptom^12^. The variety of symptoms renders diagnosis challenging; therefore, BCG osteomyelitis should be considered a possibility in young children who present with any of the aforementioned symptoms.

Three of our four cases were inoculated against TB within 1 month of birth; the fourth was vaccinated at the age of 6.5 months. According to data from the active surveillance system for BCG-associated adverse events developed by the Taiwan Centers for Disease Control, all patients with BCG-related osteomyelitis or osteitis during 2003–2012 were inoculated within 3 months after birth^13^. In 2016, Taiwan National Health Insurance program postponed the timing of vaccination to 5–8 months after birth in the hope of reducing the incidence of osteomyelitis or osteitis despite a lack of direct evidence for a connection between the timing of the vaccination and onset of condition. Consistent with previous studies^11,12^, the age at onset in our study ranged from 9 to 20 months. The time between vaccination and onset ranged from 6.5 to 19 months and had a mean duration of 11.9 months, which was similar to the 13.9 months identified in previous data in a systemic review.

Traditionally, findings on plain film for BCG osteomyelitis are characterised by osteolytic lesions and mild periosteal reaction. Typical findings were observed for all four patients in our study. However, Patient 1 had a significant periosteal reaction extending from the distal to proximal tibia. Periosteal reaction is determined by the aggressiveness, intensity, and duration of the periosteum irritation. In addition, a child with a more active periosteum may exhibit a more obvious periosteal reaction on the plain film^14^.

No standard care procedure for BCG-related osteomyelitis or osteitis has been established. According to a literature review^11^, patients who had undergone diagnostic procedures only significantly avoided major complications; thus, operations must be performed as minimally as possible. In contrast to related studies, our study involved four patients who all underwent surgical debridement. All had satisfying short-term results without residual lesions, as shown on plain film.

However, of the three patients with long-term outcomes, two developed orthopaedic complications related to BCG osteomyelitis during follow-up. One these exhibited a lesion at the right distal ulna with 0.6-cm negative ulnar variance on the most recent film. He had negative ulnar variance of 0.4 cm at his left wrist. Neither gross deformity, range of motion limitation, nor focal tenderness was observed at his most recent visit. Patient 3 underwent not only open debridement of the right distal femur but also arthroscopy with synovectomy for suspected intra-articular involvement. His affected limb had once appeared parallel Harris growth arrest line on the X-ray, and subsequently reached a length 0.92 cm longer than the other side without gait disturbance 28 months after the surgery. However, the discrepancy had decreased to 0.18 cm on the orthoroentgenogram 4 years after the initial surgery. No similar clinical course of BCG-related osteomyelitis or osteitis had been previously reported. We attributed the growth arrest and physis overgrowth to the process of recovery.

Many studies have described complications resulting from osteomyelitis in children. Evident and serious sequelae such as malalignment, joint deformity, and limb length discrepancy have long been concerns because they have a lifelong influence on function and cosmesis. Studies have documented that overgrowth is a more common consequence of increased blood supply than is undergrowth.

However, we observed undergrowth of the right upper ulna in Patient 2 and overgrowth of the right distal femur in Patient 3. We considered this phenomenon to be related to the higher ratio of lesions to physes in the distal ulna than in the distal femur, which caused relatively great damage to physes. The lesion to physis ratio of the distal ulna and femur was defined as the square of the diameter, which was 0.225 and 0.052, respectively, measured on their initial MRI. This ratio seemed a rational predictor of damage to physes. However, in Patient 4, the ratio was 0.241 but no sign of leg discrepancy was observed during the follow-up. This finding suggests that other factors, such as age, time period from disease onset to intervention, and the contribution of physes may influence growth disturbance; however, further research is required to draw a definite conclusion. Nevertheless, in our opinion, despite the influence of other factors, the distal physis of the ulna accounts for only approximately 20% of the growth, this could explain the minor discrepancy seen in our case.

In our experiences, surgery seems to be an option that does not cause harm and can be combined with medical treatment for patients who present with physis involvement. In addition, we believe that minimally invasive techniques should be applied intraoperatively to prevent harm to physes. In our study, two patients underwent gentle curettage and debridement under intraoperative fluoroscopy. In our literature review, fluoroscopy and endoscopy are used as to assist surgery to treat osteomyelitis of epiphyseal or physeal-involving lesions^15,16^. Endoscopic surgery under fluoroscopic guidance is reportedly useful for treating epiphyseal osteomyelitis caused by Mycobacterium species^17^. Intraoperative guidance with fluoroscopy or endoscopy to prevent physeal damage seems a practical solution. The physis maintains its function, and the remodeling process continues for many years.

Most recurrences of osteomyelitis occur within 6 months with initial surgery, as reported in previous studies^16,18,19^. Our patients were followed up at outpatient clinics, with radiographs taken every 2–4 weeks until bone consolidation of bone was observed on the X-rays. Radiography was then repeated every 1–2 years after the surgery if growth disturbance became a concern. Recent phone consultations revealed excellent outcomes in all four patients. This paper details the long-term outcomes of three out of the four patients. Consistent with the finding of previous studies^10–12^, their clinical manifestations seemed promising and favourable. Furthermore, we agree that monitering for serious adverse reactions following BCG vaccination and long-term outcomes is required.

Although no relevant randomised control trial has been conducted and no treatment guidelines for medical treatment for BCG osteomyelitis or osteitis have been established, combinations of treatments administered worldwide have similar foundations, including the HR regimen. Our treatment selection was consistent with a previous recommendation of a 6–12 months of HR regimen^11^. Apart from Patient 1, who is still under his HR treatment course, all patients in the present study have now completed 10–12-month HR regimens.

Several limitations of this study require further considerations. First, the rarity of BCG osteomyelitis and the retrospective nature of this study may have limited the generalisability of our observations. Although BCG detection, including molecular testing, has been provided at our national reference mycobacterial laboratory since 2008 for children aged < 5 years with isolated extrapulmonary TB strains, cases may still be misdiagnosed. In addition, the low number of cases makes further analysis impossible. Second, the patients in this study were not followed until reaching their skeletal maturity. Therefore, we are unable to determine whether deformities may develop in the future. Nevertheless, the cases in our study were confirmed through molecular methods. We demonstrated thorough clinical courses base on image findings, management, and short- and long-term outcomes. This study was conducted in a single centre with consistent treatment principles. Therefore, the patients’ clinical outcomes combined with their presentation can provide further information. Further researches on BCG-related osteomyelitis and osteitis may help us determine the role of surgical intervention, the candidates for invasive procedures, and appropriate regimens and durations of medical treatment.

In conclusion, BCG osteomyelitis and osteitis require prompt diagnosis and appropriate intervention to prevent further major complications. In cases of young children who present with mild pain and swelling of the soft tissues alongside unremarkable laboratory tests, clinicians should be alert to the possibility of skeletal TB. Timely and appropriate debridement may be crucial for patients with lesions involving physes or joint to prevent permanent sequelae.
